# The efficacy of Antipyretic Analgesics administration intravenously for Preventing Rocuronium-Associated Pain/Withdrawal Response: a systematic review and meta-analysis

**DOI:** 10.1186/s12871-020-00990-3

**Published:** 2020-04-20

**Authors:** Jia Wang, Yu Cui, Bin Liu, Jianfeng Chen

**Affiliations:** 1 West China Hospital of Sichuan University, No. 37th, Guoxue Lane, Wuhou District, Chengdu City, Sichuan Province P.R. China; 2 Chengdu Women’s & Children’s Central Hospital, Chengdu, 610000 P.R. China

**Keywords:** Rocuronium, Injection pain, Withdrawal response, Antipyretic analgesics, Meta-analysis

## Abstract

**Background:**

Rocuronium-associated injection pain/withdrawal response (RAIPWR) was non-ideal but occurred frequently when injection intravenously during anesthesia induction. Many studies had reported that pretreating with antipyretic analgesics (AAs) could reduce the occurrence of RAIPWR, but there was no consensus yet. Therefore, this meta-analysis was designed to systematically evaluate the benefits of AAs on RAIPWR in patients.

**Methods:**

PubMed, Cochrane Library, Ovid, EMbase, Chinese National Knowledge Infrastructure (CNKI), Wan Fang Data were searched by January 1st 2019 for randomized controlled trials (RCTs) applying AAs to alleviate RAIPWR in patients who underwent elective surgery under general anesthesia. Two investigators assessed quality of RCTs and extracted data respectively and the meta-analysis was carried on Revman 5.3 software. Moreover, we compared AAs in pros and cons directly with lidocaine, the most reported medicine to prevent RAIPWR.

**Results:**

Data were analyzed from 9 RCTs totaling 819 patients. The results of Meta-analysis showed that compared to the control group, pretreating with AAs could prevent the total occurrence of RAIPWR [Risk ratio (RR), 0.52; 95% confidence interval (CI), 0.42 to 0.66; *P* < 0.0001], and took effect on moderate (RR, 0.56; 95%CI, 0.43 to 0.73; *P* < 0.0001) and severe RAIPWR (RR = 0.14; 95%CI, 0.08 to 0.24; *P* < 0.00001). When compared to lidocaine, the preventive effect was not so excellent as the latter but injection pain induced by prophylactic occurred less.

**Conclusion:**

The currently available evidence suggested that pretreating with AAs intravenously could alleviate RAIPWR.

**Trial registration:**

PROSPERO CRD42019129776.

## Background

Rocuronium, a timely nondepolarizing muscle relaxant, is routinely applied in clinical anesthesia practice, and also an alternative to succinylcholine in rapid sequence induction [1] without side effects such as cardiovascular response, elevating blood potassium, or inducing myoclonus [2]. However, without preventive measures, about 50–80% [3–5] of patients experienced injection pain, and even in anesthetized patients, withdrawal movement of the arm which may soon extend to the whole body could be motivated by rocuronium injection. How to reduce the side effect of rocuronium is of significant importance of its clinical application.

Antipyretic analgesics (AAs) are a well-known category of drugs that have long been identified safe and effective to control acute postoperative pain and long-term chronic pain [6–8]. In recent years, some clinical trials reported the preventive effect of AAs on rocuronium-associated injection pain/withdrawal response (RAIPWR), but systematic review regarding the efficacy as yet has not been addressed. Thus, we aimed to assess the effectiveness of several widely used AAs of eliminating RAIPWR by conducting a Meta-analysis.

## Methods

### Source of data and search strategy

The study was conducted and presented in accordance with the systematic review guideline [9], and the study protocol was registered with the International Prospective Register of Systematic Reviews (https://www.crd.york.ac.uk/prospero/#recordDetails) with the ID of CRD42019129776. Two investigators independently searched PubMed, Cochrane Library, Ovid, EMbase, Chinese National Knowledge Infrastructure (CNKI) and Wan Fang data electronically for randomized controlled trials (RCTs) published by January 1st 2019 applying AAs to alleviate RAIPWR. Search terms included: antipyretic analgesics, acetaminophen, paracetamol, parecoxib, ketorolac, flurbiprofen, lornoxicam, rocuronium, injection pain, withdrawal. To identify all available evidence, we scanned the references cited in RCTs revolved and reviews with similar subject for eligible studies manually.

### Study selection

This system review and meta- analysis recruited RCTs meting the following criteria only:

1. Surgical patients involved in were at ASA physical status I to II and aged 2 to 75 years old.
2. Rocuronium was utilized during general anesthesia induction, and AAs were applied intravenously to prevent RAIPWR while placebo or normal saline was used in the controlled group.
3. The first outcomes of interest were the total incidence of RAIPWR and the occurrence of three degrees of RAIPWR (mild, moderate and severe). The secondary outcomes were the incidence of RAIPWR and adverse reactions of medicines used for pretreatment (AAs and lidocaine). Besides, quantitative data of outcomes were reported.
4. Outcome measurement methods: a) severity of injection pain from rocuronium was graded as follows: none, negative response to questioning; mild, pain reported in response to questioning only, without any behavioral signs; moderate, pain reported in response to questioning and accompanied by a behavioral sign, or pain reported spontaneously without questioning; severe, strong vocal response or response accompanied by facial grimacing, arm withdrawal, or tears. b) The severity of rocuronium-induced withdrawal response was rated as follows: none, no response; mild, movement at the wrist only; moderate, movement/withdrawal involving arm only (elbow/shoulder); severe, generalized response, withdrawal or movement in more than one extremity, coughing, or breath holding [5]. c) Adverse reactions of preventative medicine were evaluated by systemic (mainly cardiovascular reaction, such as hypertension, hypotension, tachycardia, bradycardia, etc.) and local reactions (the condition of injection site, such as edema, flushing or allergic reaction).
  Initially, titles and abstracts were screened to discard unrelated studies and the full text of potentially eligible studies were carefully read. Then, data on the following items would be extracted: author, published year, location of trial, type of surgery, ASA status, sample size, patient age range, type and dosage of AAs, the outcome assessment and so on. Study screening and data export were finished by two researchers respectively (Jia Wang, Jianfeng Chen), and then the works were exchanged for rationality and accuracy. If any disagreements, the third researcher (Yu Cui) would interpose and make the final decision when necessary.

### Risk of bias assessment

When an RCT met the aforementioned selection criteria, its methodological quality was assessed on the basis of the suggestions in the *Cochrane Handbook for Systematic Reviews of Interventions* [9], and the evaluation contents contained seven domains: random sequence generation(selection bias), allocation concealment (selection bias), blinding of participants and personnel (performance bias), blinding of outcome assessment (detection bias), incomplete outcome data (attrition bias), selective reporting (reporting bias) and other bias. In each special aspect of risk was graded as “yes” for low risk, “unclear” and “no” for high risk. We included a ‘Risk of bias’ detailing all of the judgements made for all included studies in the review.

### Statistical methods

Meta-analyses were carried out by Review Manager software (RevMan, version 5.3 for Windows, Oxford, UK; The Cochrane Collaboration, 2008). The categorical variable was expressed in relative risk (RR) with its 95% confidence interval (95%CI), and the continuous variable was expressed in weighted mean deviation (WMD) with 95%CI. We considered *P* < 0.05 and RR not crossing the identity line as statistically significant. Heterogeneity among studies was assessed using both the χ^2^ test and the I^2^ statistic. If *I*^2^ ≤ 50%, we considered there was no homogeneity among studies and the fixed-effects model was eligible; On the contrary, when *I*^*2*^ *>* 50%, indicating significant heterogeneity, and the random-effects model was applied for meta-analysis. In terms of outcomes with heterogeneity, an effort was made to explore the source, mainly via conducting meta-analysis stratified by patients’ characters, severity of RAIPWR and administration route of AAs, etc. We also conducted sensitivity analysis by removing studies in sequence.

## Results

### Description of studies

**Fig. 1:**
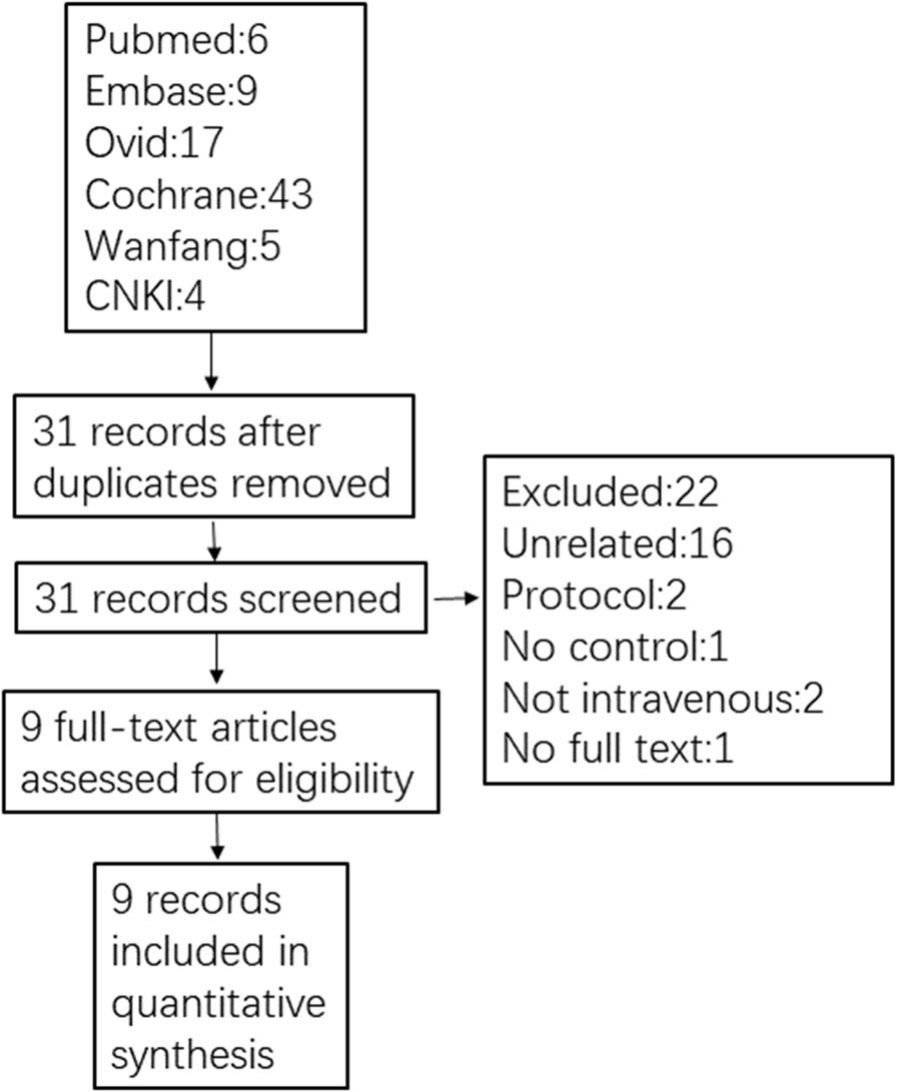
PRISMA diagram showing articles selection for this review

We initially identified 84 records according to the retrieval strategy aforementioned, and 9 [10–18] of them involving 819 patients were included eventually according to the inclusion and exclusion criteria (Fig. 1 PRISMA diagram showing article selection for this review). Six kinds of AAs (acetaminophen/paracetamol, parecoxib, ketorolac, flurbiprofen, lornoxicam and propacetamol) were reported to be used for preventing RAIPWR through two routes of intravenous administration. One was intravenous directly (IV), the other was injecting with venous occlusion (IVVO) by tourniquet. The basic characteristics of enrolled studies were listed in Table 1 (Table 1 Characteristics of studies included in Meta-analysis).

**Table 1:** Characteristics of studies included in Meta-analysis

Study	Country	Surgical setting	Age (yr)	ASA	Administration method	Group (n, patients)	Outcomes
Younghoon Jeon 2010	Korea	Elective surgery	45.4 ± 11.1 45.9 ± 14.2 50.1 ± 10.6	I-II	IVVO	N S (*n* = 39) lidocaine 40 mg (*n* = 39) paracetamol 50 mg (*n* = 40)	A/B/C/D
Yonghong Zhang 2012	China	Elective surgery	45.18 ± 12.44 41.28 ± 14.12 45.24 ± 14.36 43.54 ± 15.01	I-II	IVVO	N S (*n* = 40) lidocaine 40 mg(*n* = 40) parecoxib 20 mg(*n* = 40) parecoxib 40 mg(*n* = 40)	A/B/C/D
Younghoon Jeon 2013	Korea	Elective surgery	46.8 ± 11.5 48.5 ± 13.1 46.2 ± 16.3	I-II	IVVO	placebo(*n* = 35) lidocaine 20 mg(*n* = 35) ketorolac 10 mg(*n* = 35)	A/B/C/D
Gülnaz Ateş 2014	Turkey	Elective surgery	36.45 ± 12.94 35.58 ± 11.9 39.27 ± 11.81	I-II	IVVO	N S (*n* = 60) lidocaine 40 mg(*n* = 60) paracetamol 50 mg(*n* = 60)	A/B/C/D
Sennur Uzun 2014	Turkey	3 kinds of elective surgeries	41.8 ± 13.9 42.7 ± 11.9 41.7 ± 13.3	I-II	IVVO	N S(*n* = 50) lidocaine 40 mg(*n* = 50) aracetamol 50 mg(*n* = 50)	A/B/C/D
Cheng Yuan 2014	China	Elective surgery	45.8 ± 10.3 46.9 ± 9.6 44.3 ± 9.8 43.7 ± 10.6	I-II	IV IVVO IV IVVO	N S (*n* = 20) N S (*n* = 20) flurbiprofen 50 mg(*n* = 20) flurbiprofen 50 mg(*n* = 20)	A/B/D
Li Sha 2014	China	Elective surgery	46.7 ± 11.1 48.4 ± 13.2 46.2 ± 14.6	I-II	IVVO	N S (*n* = 35) lidocaine 40 mg (*n* = 35) flurbiprofen 50 mg(*n* = 35)	A/B/C/D
Ma Qingjie 2016	China	Elective surgery	39.2 ± 3.7 38.5 ± 4.1 40.3 ± 5.2	I	IV	N S (*n* = 40) parecoxib20mg (*n* = 40) parecoxib40mg (*n* = 40)	A/B
Yu Liang 2018	China	Elective surgery	40.7 ± 8.7 42.7 ± 7.7 43.7 ± 7.3	I-II	IV	N S (*n* = 20) lornoxicam 4 mg(*n* = 20) lornoxicam 8 mg(*n* = 20)	A/B

***NS*** normal saline, ***A*** the incidence of rocuronium-associated injection pain/withdrawal response, ***B*** the incidence of different severities rocuronium-associated injection pain/withdrawal response, ***C*** occurrence of injection pain caused by antipyretic analgesics (AAs and lidocaine), ***D*** local reaction of injection site, ***IVVO*** injection intravenously with venous occlusion, ***IV*** intravenous directedly

### Evaluation of methodological quality

**Fig. 2:**
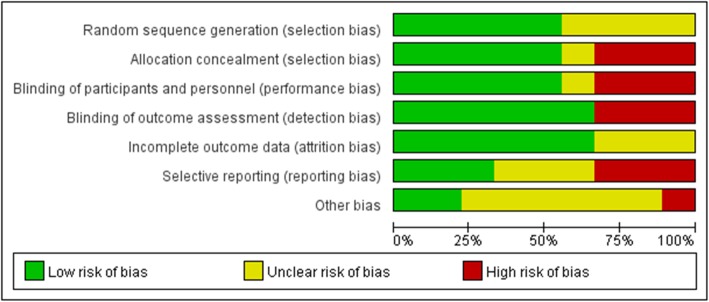
Risk of bias graph

**Fig. 3:**
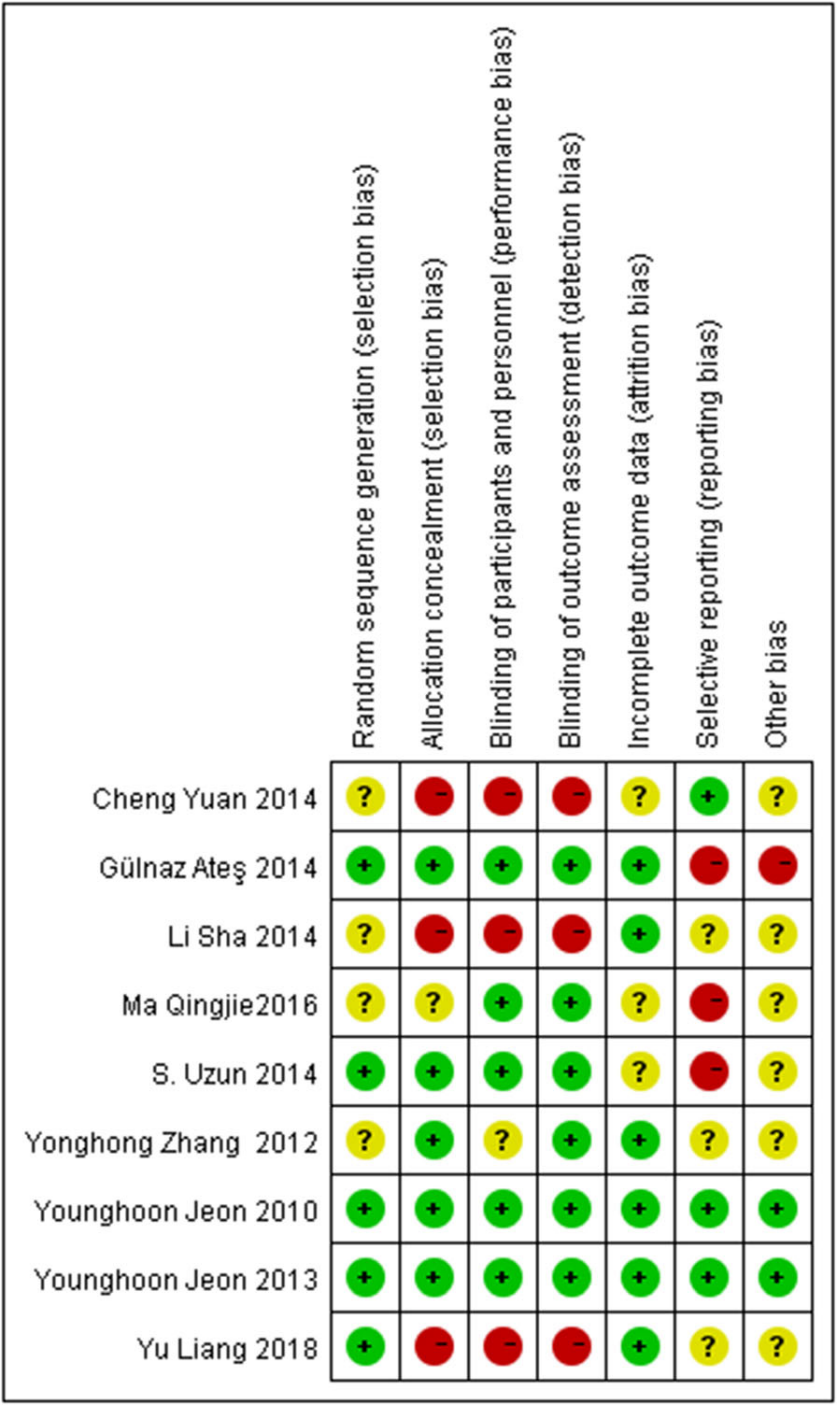
Risk of bias summary

Meticulous details regarding the risk of bias in each aspect of included studies were presented in the Risk of bias graph (Fig. 2 Risk of bias graph). Moreover, a summary of judgements about each methodological quality domain for each included RCT was shown in Fig. 3 (Fig. 3 Risk of bias summary). In general, most of studies were assessed to be of low to moderate risk of bias, and reporting bias and selective bias turned out to be the main risk of bias in this study.

### The incidence of RAIPWR

**Fig. 4:**
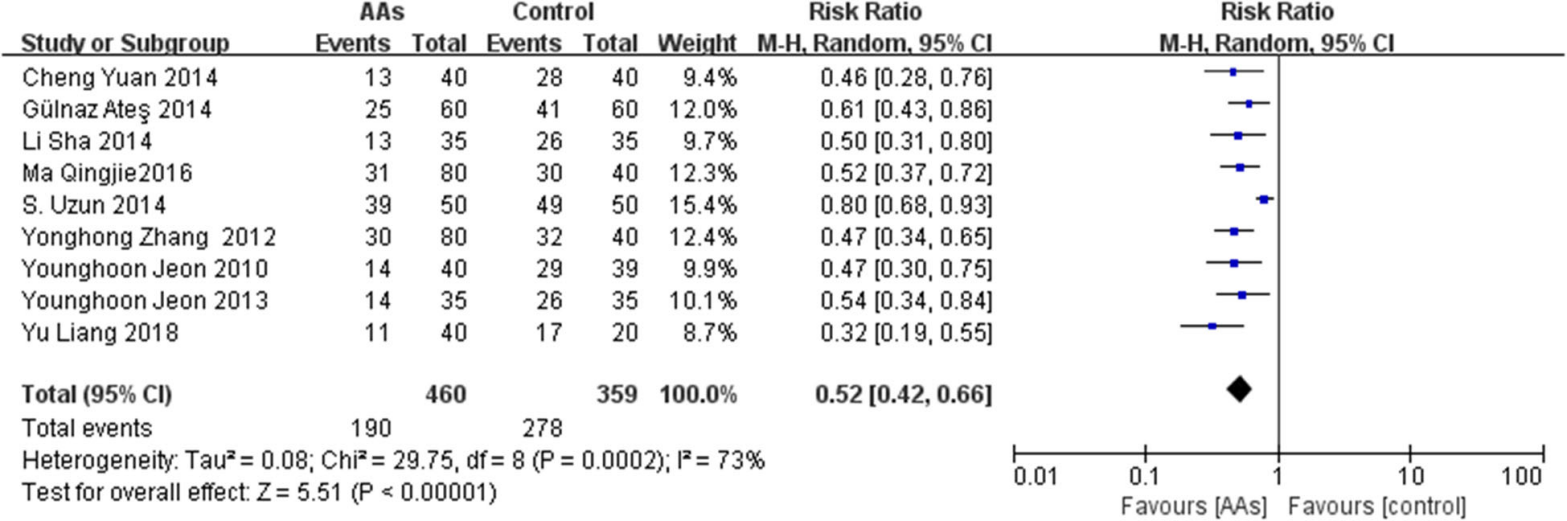
AAs vs. control-the total incidence of RAIPWR)

**Fig. 5:**
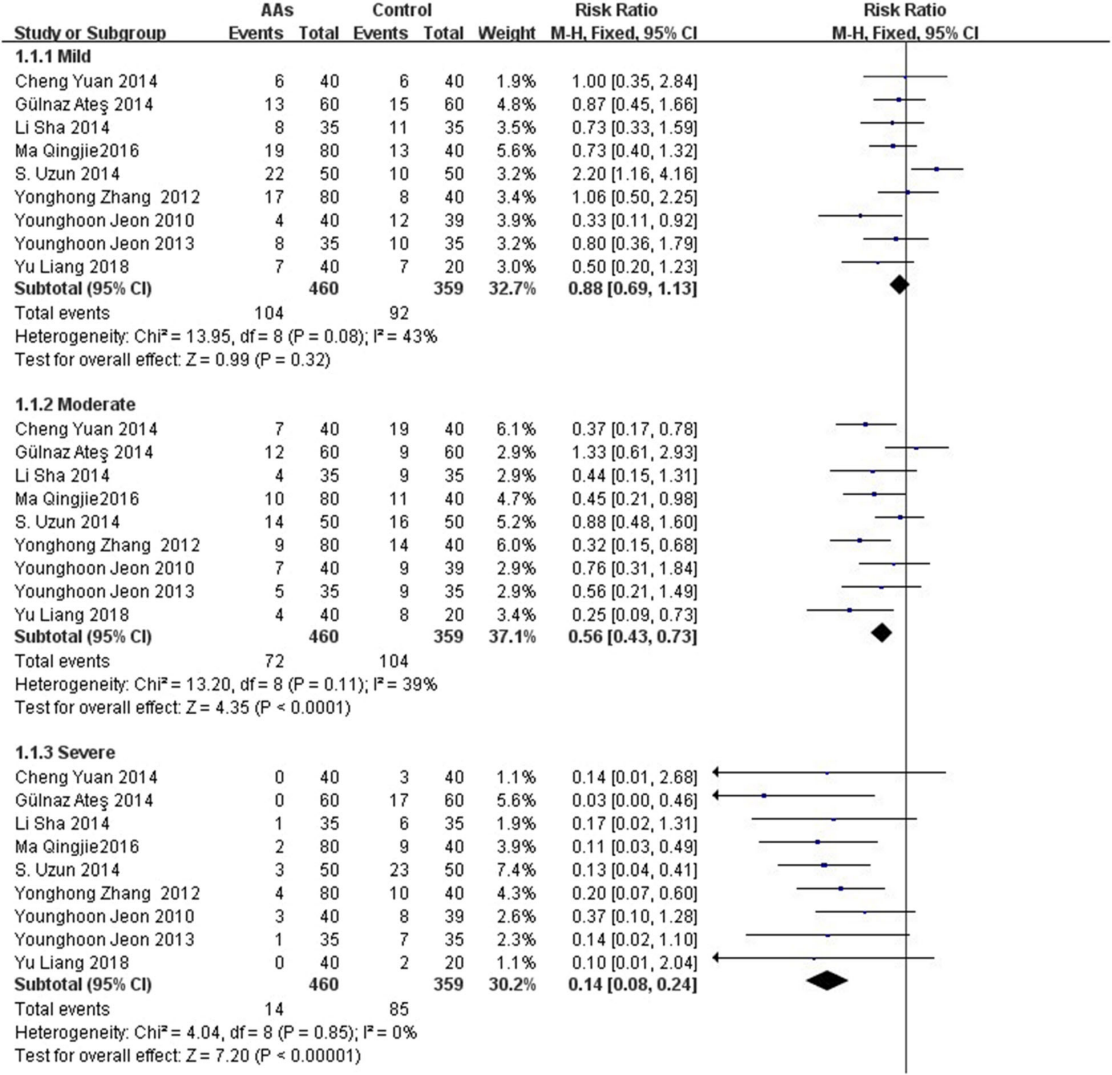
AAs vs. control-the incidence of different severities RAIPWR

**Fig. 6:**
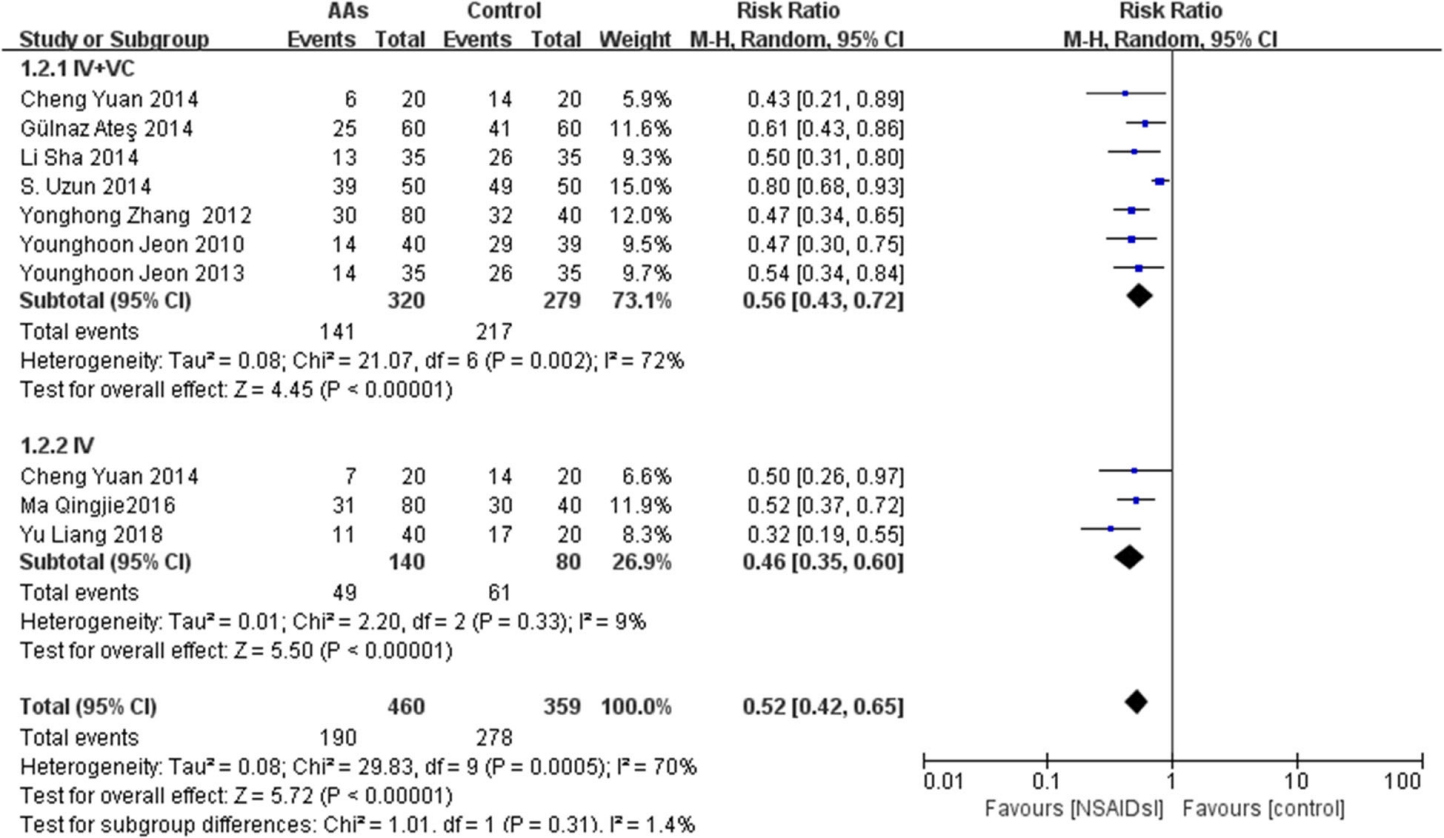
Incidence of RAIPWR-subgroup analysis of different administration methods

In this meta-analysis, 9 RCTs [10–18] with 819 patients were included and reported the incidence of total and different severities of RAIPWR. Statistical heterogeneity (*P* < 0.00001, I^2^ = 73%) was found among them, thus a random-effects model was adopted to conduct meta-analysis and the result showed the preventive effect of AAs on total RAIPWR was significant [Risk ratio (RR), 0.52; 95% confidence interval (95%CI), 0.42 to 0.66; *P* < 0.0001;I^2^ = 73%] (Fig. 4 AAs vs. control-the total incidence of RAIPWR). We further conducted subgroup analysis stratified by severity of RAIPWR and method of administration of AAs. The results which operated under fixed-effects model showed that AAs were able to drop down the incidence of moderate RAIPWR notably (RR, 0.56; 95%CI, 0.43 to 0.73; *P* < 0.0001; I^2^ = 39%) and the occurrence of severe RAIPWR (RR, 0.14; 95%CI, 0.08 to 0.24; *P* < 0.00001; I^2^ = 0%). In terms of the mild RAIPWR, AAs hadn’t shown significant effect (RR, 0.88; 95%CI, 0.69 to 1.13; *P* = 0.32; I^2^ = 43%). Generally, the results seemed to reveal that the more serious the degree of RAIPWR was, the more obvious the effect of AAs was **(**Fig. 5 AAs vs. control-the incidence of different severities RAIPWR). In addition, the results stratified by administration method of AAs indicated that AAs could reduce the incidence of RAIPWR no matter with (RR, 0.56; 95%CI, 0.43 to 0.72; *P* < 0.0001; I^2^ = 72%) or without tourniquet (RR, 0.46; 95%CI, 0.35 to 0.60; *P* < 0.00001; I^2^ = 9%) under the random-effects model (Fig. 6 Incidence of RAIPWR-subgroup analysis of different administration methods).

**Fig. 7:**
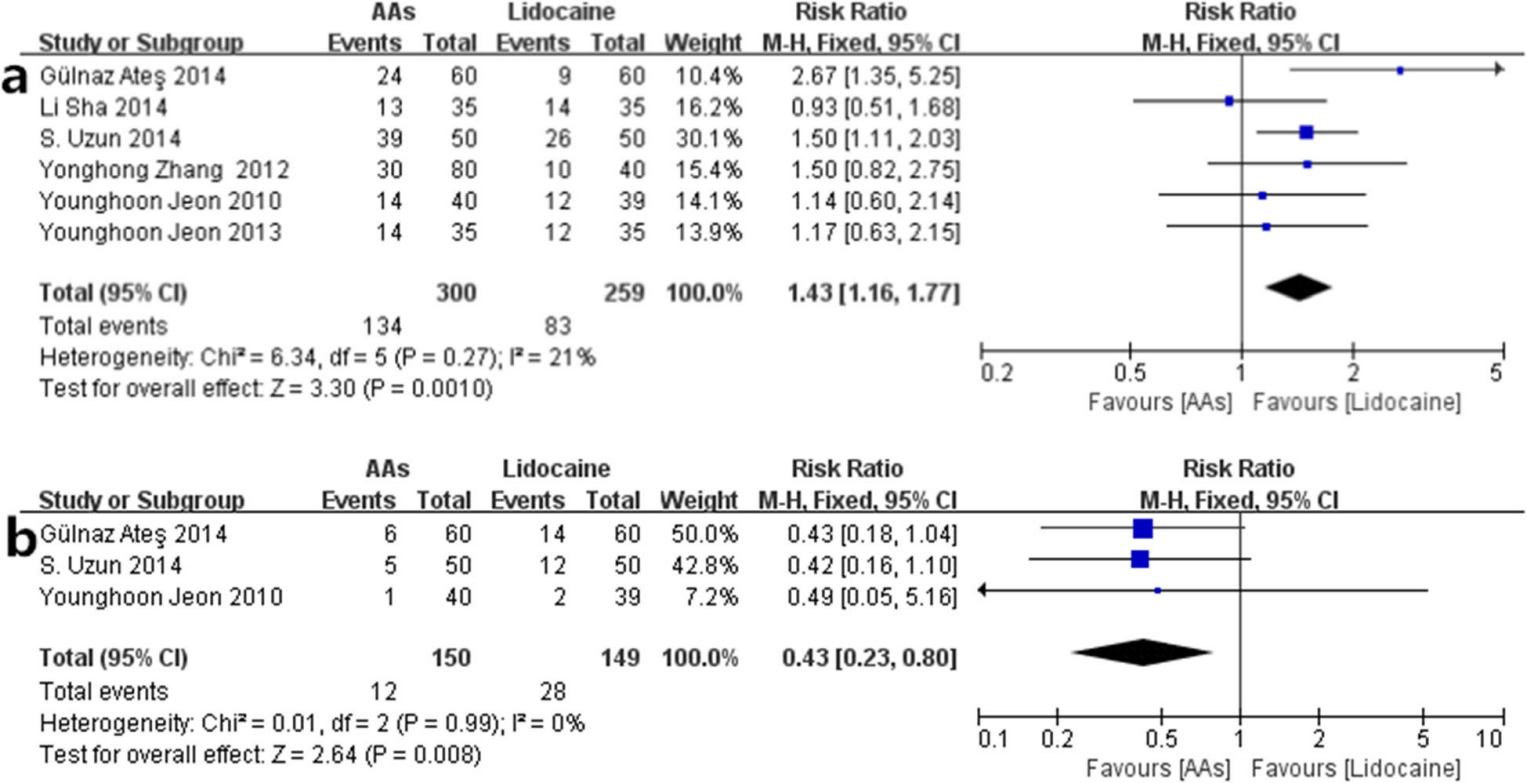
AAs vs. lidocaine- **a** the incidence of RAIPWR; **b** the incidence of injection pain from preventive drugs

*Comparison of AAs and lidocaine* (Fig. 7 AAs vs. lidocaine- **a**. the incidence of RAIPWR; **b**. the incidence of injection pain from preventive drugs).

### The incidence of RAIPWR

There were 6 studies [10–14, 16] which reported the effect of AAs and lidocaine on preventing RAIPWR. The result in the fixed-effects model showed that RAIPWR occurred more frequently in patients pretreated with AAs than lidocaine, indicating AAs were not so efficient as lidocaine on preventing RAIPWR (RR = 1.43, 95%CI (1.16, 1.77), *P* = 0.001; I^2^ = 21%). (Fig. 7a the incidence of RAIPWR).

### The side effect of AAs and lidocaine

There were 3 studies [10, 13, 14] which reported the occurrence of injection pain of prevention drugs which were used with the purpose of reducing the RAIPWR when administrated via intravenous, and no statistically heterogeneity among them (*P* = 0.99, I^2^ = 0%), so the fixed-effects model was utilized. The result suggested the incidence of injection pain of AAs was lower than that of lidocaine, and the difference was of statistically significance (RR,0.43; 95%CI, 0.23 to 0.80; *P* = 0.008; I^2^ = 0%). (Fig. 7 b the incidence of injection pain from preventive drugs).

No systemic adverse effect and skin reactions at injection site was reported.

### Sensitivity analysis

High levels of heterogeneities arose when exploring the effect of AAs on total incidence of RAIPWR and the subgroup analysis stratified by administration methods of AAs (73 and 70% respectively), and both of them disappeared when excluded one of studies [14]. Whereas, the results were consistent with that before excluding the given study in the fixed-effects model, indicating that the evaluation of corresponding effect size was stable and reliable in our Meta-analysis. (Table 2 Sensitivity analysis).

**Table 2:** Sensitivity analysis

Comparison		RR (95%CI)	P	I^2^	Effect model
Total incidence of RAIPWR	Primary analysis	0.52 (0.42,0.66)	*P* < 0.00001	73%	Random-effects
	Exclude “Uzun 2014” [14]	0.5 (0.43,0.57)	*P* < 0.00001	0%	Fixed-effects
Administration by IV + VC	Primary analysis	0.56(0.43,0.72)	*P* < 0.00001	72%	Random-effects
	Exclude “Uzun 2014” [14]	0.51(0.43,0.61)	*P* < 0.00001	0%	Fixed-effects

## Discussion

During anesthesia induction period, injection pain/withdrawal response from rocuronium occurred frequently. This meta-analysis included 9 RCTs involving 819 patients, and the observable endpoints were the incidence of total and different severities of rocuronium-induced injection pain/withdrawal response. The results indicated that AAs were effective in preventing, especially for moderate and severe RAIPWR, though not as effective as lidocaine. Our secondary outcome, pain generated by preventative medicines themselves was reported in 3 RCTs [10, 13, 14], and the result suggested the injection pain induced by AAs occurred less than that of lidocaine.

Previous studies revealed AAs were capable of alleviating injection pain from propofol [19, 20], and some studies were designed to identify the prophylactic effect of AAs on rocuronium-induced injection pain/withdrawal response under the assumption that the mechanisms of injection pain generated by propofol and rocuronium were the same. However, no conclusion has been reached on the benefit to date. Our study identified the preventive effect of AAs on RAIPWR, and compared it with lidocaine, the most widely applied pharmacological method to prevent injection pain induced by rocuronium [21], in advantages and disadvantages simultaneously, and the results indicated AAs might be more desirable for pretreatment due to less injection pain when administrated intravenously.

AAs were widely used to treat inflammatory disease for decades and with the emergence of concept of preemptive analgesia and multimodal analgesia, AAs had been a crucial part in pain management [22, 23]. As a result, when applied to prevent RAIPWR, AAs could also play a role in alleviating postoperative pain.

The mechanism of RAIPWR is still unrevealed. Arndt and Klement [24] reported that peripheral veins were invested with polymodal nociceptors, which released endogenous pain mediators such as prostaglandins after being stimulated by unphysiological osmolarity or pH of drug solution. Rocuronium has a PH of 4.0, and dilution could reduce injection pain [25], which may explain the injection pain of it [26]. Blunk and Seifert [27] demonstrated the dolorific effect of rocuronium may be on account for direct activation of C-nociceptors nerve endings with release of calcitonin prostaglandin (PG) E2 and gene-related peptide (CGRP). In animal experiments, Baek and his colleagues [28] found that rocuronium was able to suppress nitric oxide production and enhance prostaglandin E2 synthesis in calf pulmonary artery endothelial cells, inducing inflammation and pain. Antipyretic analgesics, containing nonsteroidal anti-inflammatory drugs (NSAIDs) and the most widely used analgesic in the world, acetaminophen [29], are cyclooxygenase (COX) enzyme blockers which may exert their analgesic effects via inhibiting the synthesis of prostaglandins peripherally and preventing the release of PGE2 together with activating medullary and cortical regions involved in the descending inhibitory pain cascade centrally [30].

Injecting lidocaine was reported the best intervention to prevent RAIPWR [21], and we found AAs also took effect. Though were not so effective as lidocaine, the injection pain caused by preventive medicine itself occurred less when AAs were acting as pretreatments in our review, namely, AAs may be more acceptable and suitable for patients regarding the side effect of prophylactic itself.

Limitations of our review. Firstly, our study recruited literatures published in only Chinese and English, which may lead to bias caused by the publication language. Secondly, the injection sites, dosage, injection speed of drugs and other details of pretreatment varied among enrolled studies, which may influence the results. However, all RCTs illustrated the details of the intervention: prophylactic or placebo was injected 2 to 5 min before intravenous rocuronium, and after assessment finished, other anesthetics (such as opioids and propofol) for induction were administrated, guaranteeing that the injection pain or withdrawal movement was merely caused by rocuronium, and the prophylactic effect, if any, was the result of pretreatment. Thirdly, some of studies included didn’t depict details of random sequence generation [11,15–17,] and allocation concealment [15, 16, 18], and 3 of them didn’t mention the blind method [15, 16, 18], all of above may lead to high risk of bias, so the power of this review was confined. We discovered Uzun’s study [14] was the main source of heterogeneity when carried out sensitive analysis, so we rechecked this study, and found that methodology it abided by resembled to others but they enrolled patients who underwent elective orthopedic, gastrointestinal, and gynecological procedures while other studies recruited all kinds of elective surgeries. Given the impossible task of conducting subgroup analysis stratified by operation types, and the results were homogeneous when excluded the particular study or not, we didn’t do further analysis.

## Conclusion

In this meta-analysis, current evidence suggested that pretreating with AAs was effective in dropping the occurrence of the RAIPWR, and especially in term of moderate and severe degree of it. However, comparing to pretreating with lidocaine, AAs were not so efficient as the latter, while caused less injection pain and might be more suitable for pretreatment. Considering the quality and quantity of studies involved in this review, it was recommended that more multicenter, randomized, and double-blind controlled trials with larger samples size were needed to confirm the above conclusions.

## Data Availability

The analyzed datasets generated during the study are available from the corresponding author on reasonable request.

## Abbreviations

RAIPWR: Rocuronium-associated injection pain/withdrawal response
AAS: Antipyretic analgesics
ASA: American Society of Anesthesiology
RCT: Randomize control trial
CI: Confidence interval
NSAIDs: Nonsteroidal anti-inflammatory drugs
COX: Cyclooxygenase
GRP: Gene-related peptide
NS: Normal saline
IVVO: Injection intravenously with venous occlusion
IV: Injection intravenously
